# Peritumor tertiary lymphoid structures are associated with infiltrating neutrophils and inferior prognosis in hepatocellular carcinoma

**DOI:** 10.1002/cam4.5227

**Published:** 2022-09-09

**Authors:** Tianchen Zhang, Xinjun Lei, Weili Jia, Jianhui Li, Ye Nie, Zhenzhen Mao, Yanfang Wang, Kaishan Tao, Wenjie Song

**Affiliations:** ^1^ Department of General surgery The First Affiliated Hospital of Xi'an Medical University Xi'an Shaanxi China; ^2^ Department of Hepatobiliary Surgery Xijing Hospital, The Fourth Military Medical University Xi'an Shaanxi China

**Keywords:** hepatocellular carcinoma, infiltrating neutrophils, tertiary lymphoid structures, tumor prognosis

## Abstract

**Background:**

The positive prediction of prognosis and immunotherapy within tertiary lymphoid structure (TLS) in cancerous tissue has been well demonstrated, including liver cancer. However, the relationship between TLS and prognosis in the peritumoral region of hepatocellular carcinoma (HCC) has received less attention. Few studies on whether TLS, as a typical representative of acquired immune cell groups, is associated with innate immune cells. The aim of this paper was to identify the prognostic role of peritumor TLS in HCC and to simply explore the relationship with neutrophils infiltration.

**Methods:**

This study included cancerous and paracancerous tissue from 170 patients after surgical resection of HCC. TLS was examined and identified by pathological H&E examination, and the impact on prognosis was further classified by determination of total TLS area. Immunohistochemical staining of CD15+ neutrophils was also performed on half of the cases. The obtained results were validated by external public database, as TLS has been widely shown to be tagged with 12 chemokines.

**Results:**

In peritumoral tissue, the TLS− group had better overall survival (OS) and disease‐free survival (DFS) outcomes compared with the TLS+ group. On the contrary, the intratumor TLS+ group showed better DFS outcomes. When further investigating the relationship between TLS area distribution and DFS, progressively worse prognosis was only found in the peritumor region with increasing TLS density (TLS− vs. TLS_L_ vs. TLS_H_). In addition, neutrophil infiltration increased in parallel with TLS density in the peritumoral region, which was not observed in the intratumoral region.

**Conclusions:**

TLS might have a dual prognostic role in different regions of HCC. The abundance of peritumoral TLS is an independent influence of DFS. The inconsistent correlation between neutrophils and corresponding TLS in different regions may indicate different pathways of immune aggregation and may serve as an explanation for the different prognosis of TLS, which needs to be specifically explored.

## Graphical abstract

The positive role of tertiary lymphoid structure in tumor has received much attention, but peritumoral tertiary lymphoid structure in Hepatocellular Carcinoma has a different picture, which may be associated with neutrophil infiltration.

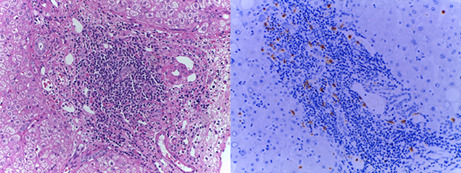

## 1 INTRODUCTION

Liver cancer is the sixth most frequent cancer globally and the third leading cause of tumor‐related deaths.^1^ Hepatocellular carcinoma (HCC) is the most common primary liver cancer, accounting for 75%–85% of cases, and there are significant regional differences in its pathogenic factors. The main risk factors worldwide were hepatitis B virus (HBV, 33%), alcohol (30%), hepatitis C virus (HCV, 21%), and others (16%).^1^, ^2^ In HCC, surgery remains the treatment with the greatest survival benefit, but the recurrence rate is as high as 80% at 5 years after surgery.^3^ With the advancement of tumor microenvironment (TME) research, the treatment of malignant tumors has entered a new era of immunotherapy and has benefited a lot. For example, immune checkpoint inhibitors have the greatest benefit in melanoma and Hodgkin lymphoma with an ultra‐high response rate of 70%–87%, while the objective response rate in HCC is only 20%.^4^

Tertiary lymphoid structures, also known as ectopic lymphoid organs, are structurally and functionally similar to secondary lymphoid organs and present in chronic inflammation, autoimmune diseases, organ transplantation, and tumors.^5^ Mature TLS is composed of a T‐cell zone containing dendritic cells and a B‐cell follicular zone with a germinal center, within which high endothelial venule, a nutrient and lymphocyte transport duct, is scattered.^6^ In addition, studies have revealed the presence of both anti‐tumor and pro‐tumor immune cells in the TLS, such as CD8+ T cells, plasma cells, macrophages, regulatory T cells (Treg), myeloid‐derived suppressor cells (MDSC) and so on.^7^, ^8^, ^9^ Being a more direct and extensive site for anti‐tumor immune responses in the TME, the aggregated immune cells in TLS are more easily stimulated by tumor antigens and cytokines than SLO, due to the lack of fibrous capsules outside the TLS.

Therefore, TLS has been widely focused in the era of immunotherapy for abundant immune cell aggregation. In fact, TLS has been shown to represent a good prognosis and good immunotherapeutic effect in many solid tumors, such as melanoma, sarcoma, colorectal cancer, and ovarian cancer,^10^, ^11^, ^12^, ^13^ including HCC.^14^, ^15^ However, TLS in the paracancerous region of HCC has received less attention and is controversial in terms of prognostic outcome. Finkin et al. believed that TLS had a poor prognosis and found that TLS first appeared in malignant progenitor cells in mice induced.^16^ However, Lihui et al. found that peritumor TLS represented a good prognosis after scoring TLS density.^17^

In this paper, 170 HCC cancer and paracancerous pathological sections were explored for prognostic research, and the total area classification method was used for the first time to more comprehensively evaluate TLS density. The correlation between TLS as a collection of adaptive immunity and neutrophils as a symbol of innate immunity and rapid inflammatory response cells is also briefly explored.

## 2 MATERIAL AND METHODS

### 2.1 Patients and samples

The training cohort (“XJ cohort”) included 170 patients who underwent curative resection of HCC at XiJing Hospital Affiliated to Air Force Military Medical University from 2016 to 2018. We reviewed and documented the following clinical and biological features: age, gender, smoking history, drinking history, hepatitis B virus (HBV) infection, cirrhosis, preoperative alpha‐fetoprotein (AFP) serum level, single tumor nodule, tumor size, tumor differentiation, capsule, and microvascular invasion. Tumor stages were evaluated using the tumor‐node‐metastasis staging system (TNM, 8th edition). For the evaluation of liver function, we used the terms of “ALBI,” which is calculated based on total bilirubin and albumin, and does not contain subjective indicators compared with CHILD classification.^18^ This study was approved by the Institutional Ethics Committee and informed consent was obtained prior to surgery for the use of surgical specimens and associated clinical data.

The validation cohort (“TCGA cohort”) was derived from 50 paracancerous data and corresponding cancer data in the the cancer genome atlas (TCGA) database. We used the data after normalization of the transcriptome, which the biometric variables included: age, gender, HBV, HCV, AFP, and TNM stage. The main outcome events for the prognosis in this study were overall survival (OS) and disease‐free survival (DFS). OS was calculated from the date of liver resection to death or, in those alive, to the date of the last follow‐up. DFS was defined as the time from surgical resection to recurrence or progression, death from any cause, or the last follow‐up.

### 2.2 Pathological examination

**FIGURE 1:**
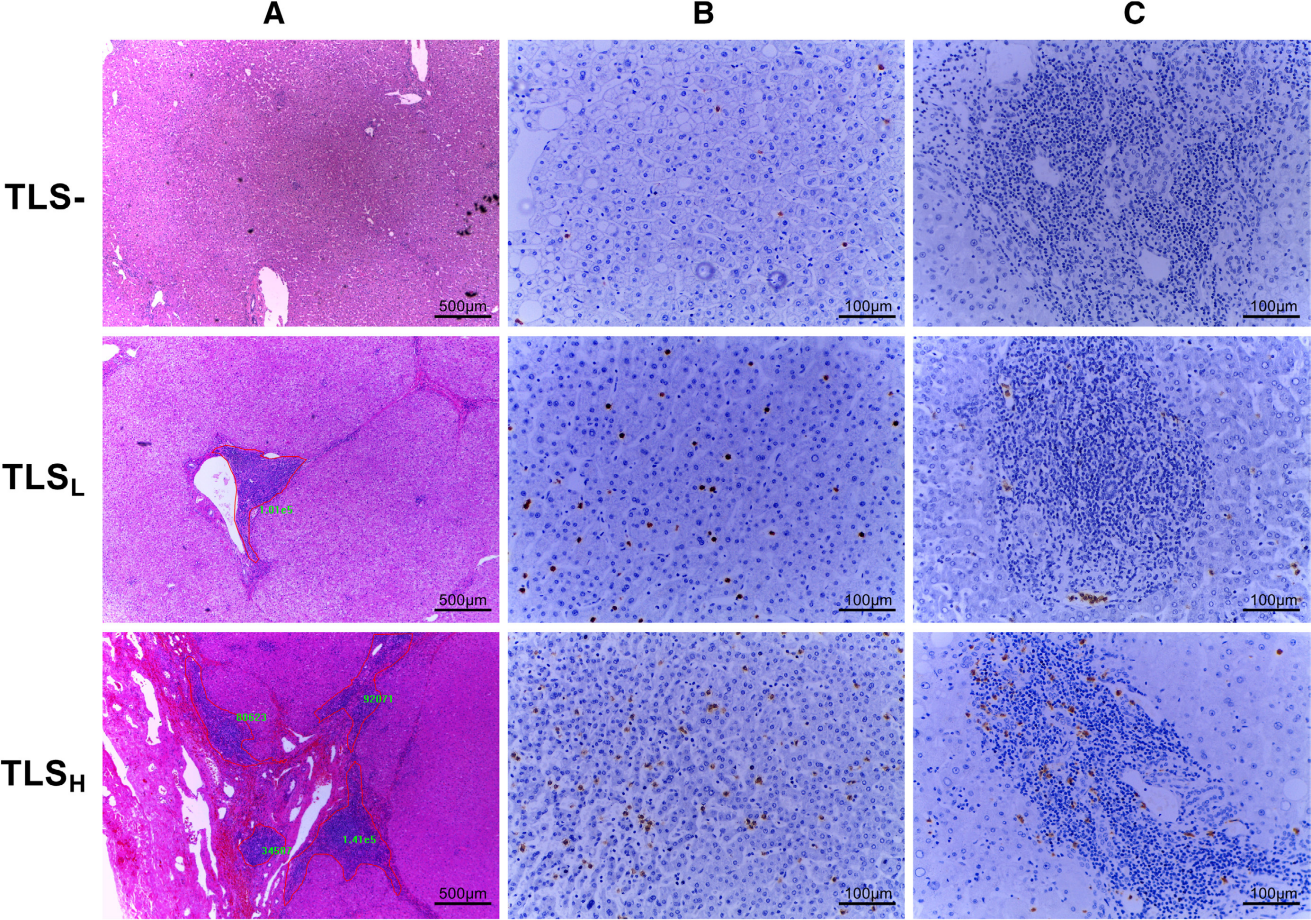
Histological appearance of tertiary lymphoid structure and neutrophil infiltration in peritumor. (A) TLS was recognized by H&E (X40). TLS−, No aggregate in whole slide; TLS_L_ and TLS_H_ distinguished by the cut‐off of total area of immune aggregation. (B) Neutrophil infiltration in the corresponding three groups (X200). (C) Irregular distribution of neutrophils within the TLS, even negative (X200). TLS, tertiary lymphoid structure; TLS_H_, TLS high; TLS_L_, TLS low.

Serial sections of surgically resected tumor paraffin masses, including intratumoral area and peritumoral parenchyma, were used for histological evaluation. The peritumoral tissue was obtained at a distance of 1 cm from the tumor edge, and there was no tumor visible under the naked eye and microscope, because the surgery was performed with R0 resection. Two sections were taken for hematoxylin and eosin (H&E) staining and judged by two experienced pathologists. Result determination: the complete absence of TLS in the whole section was determined as TLS−, and the others were TLS+. To further investigate the prognostic value of the TLS area gradient, all TLS‐positive regions were photographed (40x mirror, pixels and exposure conditions were standardized). The TLS total area was calculated as the sum of each TLS measured by the manual box selection function of the software Image Pro Plus (Figure 1A). The minimum *p*‐value method was used to further divide TLS+ into two groups,^19^, ^20^ the TLS low group (TLS_L_) and the TLS high group (TLS_H_). In our work, the cut‐off values of peritumor and intratumor are 109,941 pixels and 59,440 pixels, respectively.

### 2.3 Immunohistochemistry (IHC)

Half of the XJ cohort were randomly selected according to the original tissue proportion of the peritumor TLS subgroups (TLS–, TLS_L_, and TLS_H_). The obtained 85 samples and their corresponding cancer tissues were sectioned. Accordingly, 85 pairs of cancerous and paracancerous pathological slides were used to assess neutrophil infiltration by CD15 immunohistochemical staining (Anti‐CD15[FUT4/815], ab212396, abcam), and CD15 dilution used 1:1000. Five fields (200× high‐power) per area were evaluated for immune markers. The number of stained cells (except neutrophils in the necrotic area) per 200× high‐power field was counted (cell number).^21^ Neutrophil counts were also performed independently by two pathologists and designed as a double‐blind trial without knowledge of TLS and clinical information in the patients. At the same time, it was found that the discrimination of TLS had a high consistency with H&E.

### 2.4 Gene expression analysis

TLS was determined by the ssGSEA enrichment scores of 12 chemokines (CCL2, CCL4, CCL5, CCL8, CCL18, CCL19, CCL21, CXCL9, CXCL10, CXCL11, and CXCL13).^22^, ^23^ Based on the different proportions of TLS in different regional tissues of HCC in previous studies,^14^, ^15^, ^17^ we classified those scoring inferior to the one quartile in the paracancer as peritumor TLS− (PTLS−), and the score superior to the third quartile in the cancer as intratumor TLS+ (ITLS+). FUT4 gene expression corresponding to the antibody we used in IHC was selected as the observation index of neutrophil infiltration.

### 2.5 Statistical analysis

All the statistical analyses were performed by applying SPSS software (version 26.0, SPSS Inc., Chicago, IL, USA), GraphPad Prism software (version 8.0, La Jolla, CA, USA), Image Pro Plus (version 6.0), and R software (version 4.0). Categorical variables were compared using the chi‐square test or Fisher's exact test. Continuous variables were compared using the *t*‐test, Mann–Whitney *U* test or Kruskal–Wallis rank test. Survival curves were drawn using the Kaplan–Meier method and compared using the log‐rank test. Multivariate analysis used a Cox proportional hazards regression model. Spearman analysis was used to test the correlation of skewed data. Statistical results *p* < 0.05 was considered to be statistically significant.

## 3 RESULTS

### 3.1 XJ cohort

#### 3.1.1 Peritumor TLS with negative prognosis

Patients were male and older than 55 years in 85% and 48% of the training cohort, respectively (Table 1). The main risk factors were HBV (*n* = 127, 75%), alcohol intake (*n* = 33, 19%), as HCV was not included in the study for there were only six samples. TNM stage was classified as very early (I, *n* = 56, 33%), early (II, *n* = 84, 49%), intermediate (III, *n* = 26, 15%), and advanced (IV, *n* = 4, 2%). ALBI score, a grade for universal assessment of hepatic function, were identified separately grade 1(*n* = 130), grade 2(*n* = 39), and grade 3(*n* = 1).

**TABLE 1:** Clinical and biological features of the XJ cohort according to the presence of PTLS

Variables	*n* = 170	TLS– *n* = 28(17%)	TLS_L_ *n* = 75(44%)	TLS_H_ *n* = 67(39%)	*p* value
Age (years)					
>55	81	15(18.5%)	34(42%)	32(39.5%)	0.76
≤55	89	13(14.6%)	41(46.1%)	35(39.3%)	
Gender					
Male	143	22(15.4%)	59(41.3%)	62(43.4%)	0.05
Female	27	6(22.2%)	16(59.3%)	5(18.5%)	
Smoking					
Yes	64	8(12.5%)	24(37.5%)	32(50%)	0.09
No	106	20(18.9%)	51(48.1%)	35(33%)	
Alcohol					
Yes	33	7(21.2%)	10(30.3%)	16(48.5%)	0.20
No	137	21(15.3%)	65(47.4%)	51(37.2%)	
HBV infection					
Yes	127	22(17.3%)	55(43.3%)	50(39.4%)	0.86
No	43	6(14%)	20(46.5%)	17(39.5%)	
Cirrhosis					
Yes	113	21(18.6%)	45(39.8%)	47(41.6%)	0.26
No	57	7(12.3%)	30(52.6%)	20(35.1%)	
AFP (ng/ml)					
>300	59	8(13.6%)	25(42.4%)	26(44.1%)	0.60
≤300	111	20(18%)	50(45%)	41(36.9%)	
ALBI grade					
I	39	4(10.3%)	14(35.9%)	21(53.8%)	0.10
II/III	131	24(18.3%)	61(46.6%)	46(35.1%)	
TNM stage					
I/II	28	4(14.3%)	13(46.4%)	11(39.3%)	0.93
III/IV	142	24(16.9%)	62(43.7%)	56(39.4%)	
Tumor number					
Single	25	6(24%)	8(32%)	11(44%)	0.34
Mutiple	145	22(15.2%)	67(46.2%)	56(38.6%)	
Tumor size (cm)					
>5	91	13(14.3%)	41(45.1%)	37(40.7%)	0.71
≤5	79	15(19%)	34(43%)	30(38%)	
Differentiated degree					
Poor	24	5(20.8%)	12(50%)	7(29.2%)	0.53
Middle/high	146	23(15.8%)	63(43.2%)	60(41.1%)	
Capsular					
Yes	141	20(14.2%)	62(44%)	59(41.8%)	0.14
No	29	8(27.6%)	13(44.8%)	8(27.6%)	
MVI					
Yes	86	15(17.4%)	39(45.3%)	32(37.2%)	0.83
No	84	13(15.5%)	36(42.9%)	35(41.7%)	

*Note*: Statistical analysis was performed using chi‐square tests.

Abbreviations: AFP, alpha‐fetoprotein; ALBI, albumin‐bilirubin; HBV, hepatitis B virus; MVI, microvascular invasion; PTLS, peritumoral tertiary lymphoid structure; TLS, tertiary lymphoid structure; TNM, tumor‐node‐metastasis.

Pathological examination showed the absence of TLS in 28 peritumor (17%) (Table 1). Among peritumor TLS+, TLS_L_ (*n* = 75, 53%), and TLS_H_ (*n* = 67, 47%) were grouped by the total area of TLS, a way to represent density abundance (Figure 1A). The grade of TLS area was not found to be associated with any clinical, biological, or tumor characteristics (Table 1).

We recorded totally 56 recurrences and 69 deaths, of which 33 died with relapses. The univariate analyses of OS and DFS are shown in Table 2. Features associated with an increased risk of both OS and DFS were HBV, AFP serum level, ALBI score, tumor stage, tumor size and the state of TLS, except that Capsular (HR = 0.53, *p* = 0.03) was the protect factor for OS in Univariate Cox regression analysis. Univariate analysis then revealed that poor differentiation (*p* value = 0.5455 and 0.9241 for OS and DFS, respectively) and microvascular invasion (*p* value = 0.0798 and 0.1214 for OS and DFS, respectively) were not correlated with OS and DFS.

**TABLE 2:** Analysis of OS and DFS in PERITUMOR of the XJ cohort

Variables	Overall survival				Disease‐free survival			
	Univariate analysis		Multivariate analysis		Univariate analysis		Multivariate analysis	
	HR (CI 95%)	*p* value	HR (CI 95%)	*p* value	HR (CI 95%)	*p* value	HR (CI 95%)	*p* value
Male sex	2.13(0.92–4.92)	0.08			1.96(0.98–3.89)	0.06		
Age > 55 years	0.62(0.38–1)	0.05			0.69(0.46–1.05)	0.08		
Smoking	1.35(0.84–2.17)	0.22			1.4(0.93–2.12)	0.11		
Alcohol	1.48(0.86–2.56)	0.16			1.32(0.8–2.17)	0.27		
HBV	2.27(1.16–4.45)	0.02	2.11(1.06–4.2)	0.03	1.88(1.11–3.18)	0.02	1.72(1–2.97)	0.05
Cirrhosis	1.26(0.75–2.12)	0.38			1.26(0.81–1.98)	0.31		
AFP > 300 (ng/ml)	1.67(1.03–2.69)	0.04	1.54(0.95–2.52)	0.08	1.62(1.06–2.46)	0.03	1.43(0.94–2.2)	0.10
ALBI grade (2/3)	2.2(1.33–3.64)	0.002	1.66(0.97–2.84)	0.06	2.27(1.47–3.53)	<0.001	1.68(1.06–2.66)	0.03
TNM stage (III/IV)	3.06(1.79–5.22)	0.000	1.87(0.91–3.84)	0.09	2.25(1.36–3.72)	0.002	1.69(0.98–2.9)	0.06
Nodosity	1.22(0.64–2.32)	0.55			1.22(0.69–2.16)	0.49		
Tumor size (>5 cm)	2.18(1.31–3.61)	0.003	1.69(0.97–2.95)	0.06	1.93(1.26–2.96)	0.003	1.48(0.92–2.37)	0.11
Poor differentiation	1.23(0.63–2.41)	0.55			1.03(0.56–1.89)	0.92		
Capsular	0.53(0.3–0.95)	0.03	0.7(0.33–1.47)	0.34	0.63(0.37–1.08)	0.09		
MVI	1.54(0.95–2.49)	0.08			1.39(0.92–2.1)	0.12		
TLS	3.53(1.28–9.7)	0.01	3.65(1.29–10.36)	0.02	3.03(1.4–6.56)	0.005	–	–
Dimension classification		0.03	–	–		0.001		0.002
TLS–	0.38(0.16–0.9)	0.009			0.25(0.11–0.56)	0.001	0.28(0.12–0.62)	0.002
TLS_L_	0.79(0.48–1.28)	0.33			0.59(0.39–0.91)	0.017	0.6(0.39–0.93)	0.02
TLS_H_	reference				reference		reference	

*Note*: Statistical analysis was performed using univariate and multivariate Cox proportional hazards regression models.

Abbreviations: AFP, alpha‐fetoprotein; ALBI, albumin‐bilirubin; DFS, disease‐free survival; HBV, hepatitis B virus; HR, hazard ratio; MVI, microvascular invasion; OS, overall survival; TLS, tertiary lymphoid structure; TNM, tumor‐node‐metastasis; –, using the other variables (TLS/Dimension classification).

**FIGURE 2:**
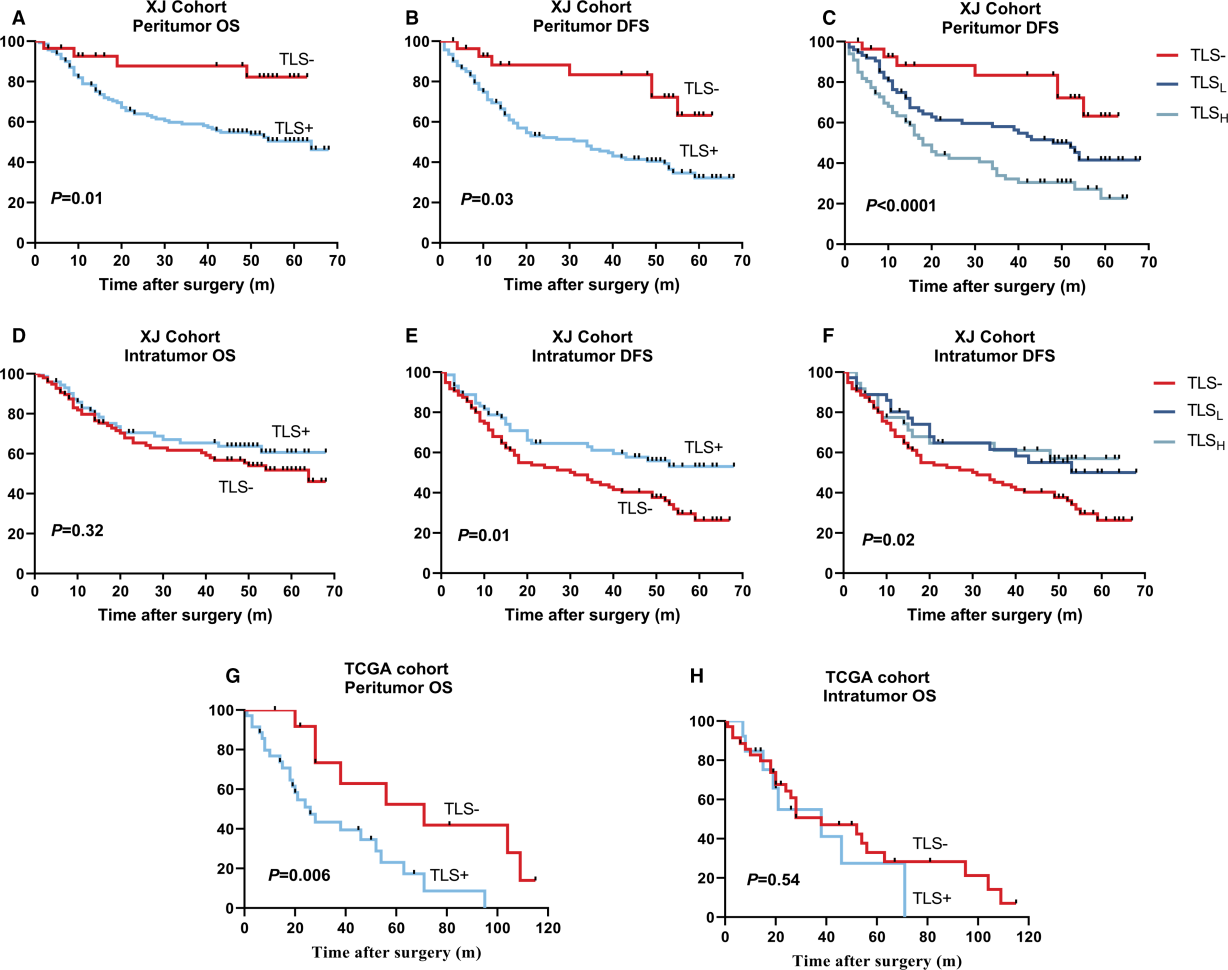
Impact of tertiary lymphoid structures on OS and DFS in both cohorts. (A, B, G) Patients with peritumor TLS display a lower survival in training and validation cohort. (C) The risk of DFS is different according to the dimension classification of Peritumor TLS (either TLS− or TLS_L_ vs. TLS_H_, *p* < 0.0001). (D, H) Intratumor TLS has no impact on the risk of OS in both cohorts. (E) But, Patients with intratumor TLS show a superior DFS in XJ cohort. (F) No different risk of DFS found in grade of intratumor TLS. DFS, disease‐free survival; OS, overall survival; TLS, tertiary lymphoid structure; TLS_H_, TLS high; TLS_L_, TLS low.

Factors with significant differences by univariate survival analysis were entered into a multivariate (Cox proportional hazards) regression model. Peritumor‐positive TLS was found as an independent and inferior prognostic factor in both OS (HR = 3.65, *p* = 0.02) and DFS (HR = 2.76, *p* = 0.01) (Table 2 and Figure 2A, B). Moreover, the grade of PTLS dimension (TLS− vs. TLS_L_ vs. TLS_H_, *p* = 0.002) was significantly associated with DFS notably (Table 2 and Figure 2C).

#### 3.1.2 Prognosis of intratumor TLS

Apart from an association with large tumor size (>5 cm, *p* = 0.02) and positive hepatic B virus (*p* = 0.009), TLS− was not linked to any other clinical, biological, or pathological features (Table S1). The prognostic role of intratumor TLS in HCC is same as previous study in PAN‐CANCER, as expected. Interestingly, ITLS+ remained significantly related to DFS (*p* = 0.02) but not OS (*p* = 0.32) (Figure 2D, E), whereas TLS_L_ and TLS_H_ in intratumor have not found significance in DFS unlike peritumor (Figure 2F).

#### 3.1.3 TLS and neutrophils in both region

We next investigated the relationship between TLS and infiltrating neutrophils abundance in both intratumor and peritumor section. The clinical features of group, half of the XJ cohort, were shown in Table S2.

**FIGURE 3:**
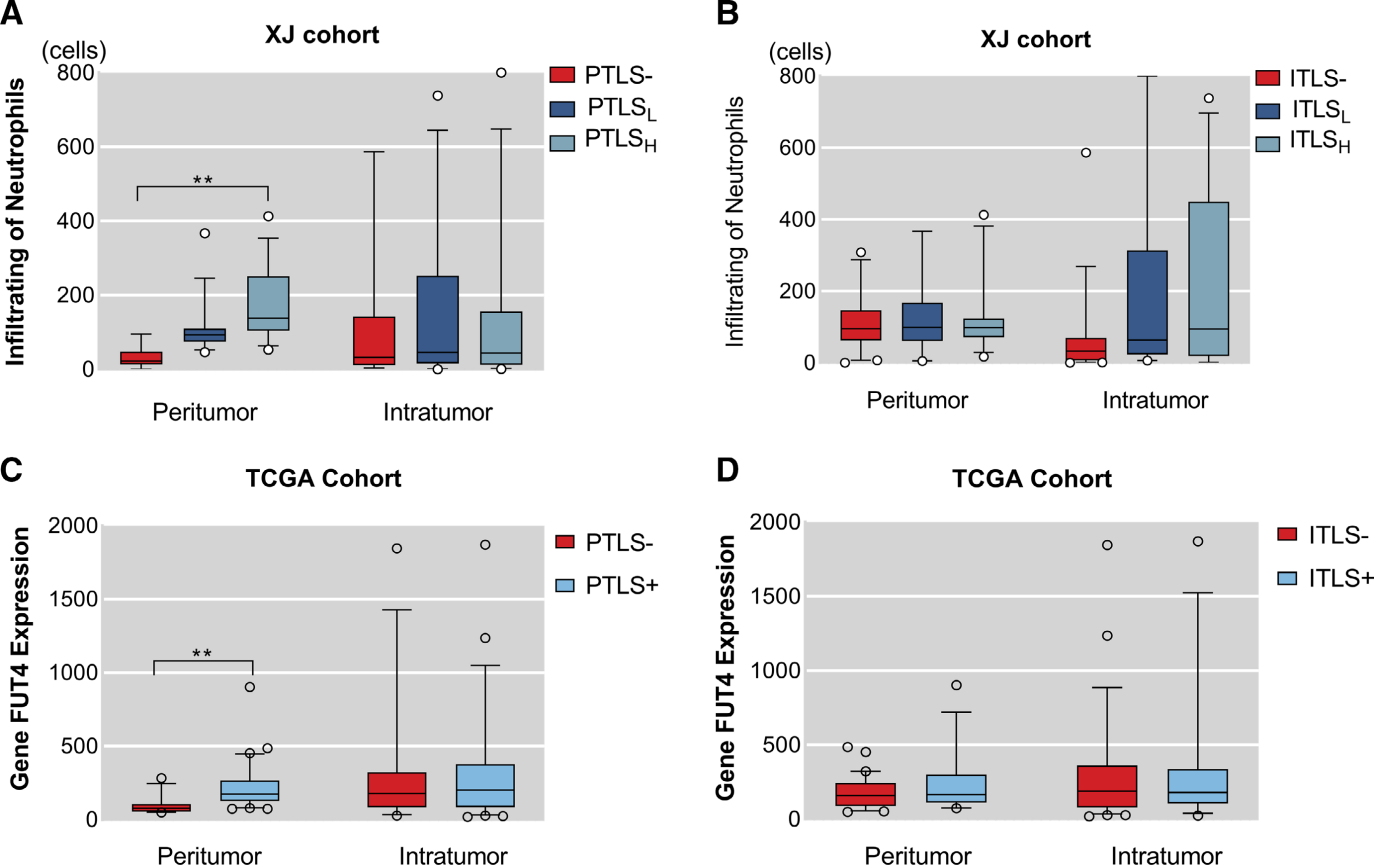
Association of TLS and Neutrophils in both cohorts. (A, B) In XJ cohort, neutrophil infiltration in paraneoplastic tissue is positively correlated with PTLS grade, but not with ITLS grade. No difference in neutrophils infiltration in cancer tissue was found in two types of TLS. (C, D) The validation cohort confirmed the correlation between neutrophils and TLS in peritumor. ITLS, intratumor tertiary lymphoid structure; PTLS, peritumor tertiary lymphoid structure; *p < 0.05; **p < 0.001.

With increasing grade of PTLS, the number of infiltrating neutrophils in both areas were peritumor (mean rank = 9.71, 30.95, 60.03, *p* < 0.001) and intratumor (mean rank = 38.68, 45.3, 42.28, *p* = 0.68), which P values are from the Kruskal–Wallis rank test. (Figure 3A). However, no statistically significant change in neutrophil infiltration which belongs peritumor (mean rank = 42.82, 44.05, 42.52, *p* = 0.98) and intratumor (mean rank = 34.44, 50.89, 49.81, *p* = 0.013) was found in elevated grade of ITLS (Figure 3B). In fact, the number of neutrophils was linked to the positive TLS in tumor (ITLS− vs. ITLS+, mean rank = 34.4 vs. 50.2, *p* = 0.003) (Figure 3B).

No meaningful results were found in the study of neutrophils distribution within the TLS, with varying degrees of oligo‐infiltrating and rich‐diffusion in different TLS that present in the same tissue (Figure 1C).

### 3.2 TCGA cohort

To validate our overall findings, the relationship between peritumor TLS and prognosis, and the association of TLS with neutrophil infiltration in different subregions, we further analyzed data from public database TCGA, which consists of 50 individuals with HCC treated by surgical resection. Since only one relapsed in the entire cohort, we only performed the validation of OS.

Compared with XJ cohort, this group was older, 68% were over 55 years old, but the HBV infection rate decreased to only 43% (Table **3**). Peritumor TLS+ was not associated with any clinical or biological variables. Univariate analysis of OS: male (HR = 4.02, *p* = 0.002), TLS (HR = 3.35, *p* = 0.009). However, multivariate analysis further revealed that PTLS (HR = 2.36, *p* = 0.08) (Table S3) was not an independent prognostic risk factor, probably due to the small total sample size and observed variables. When the Log‐Rank test was performed on the OS survival curve, PTLS+ also showed inferior prognosis (*p* = 0.006) (Figure 2G), and no significance was found in OS when focus on TLS of intratumor (Figure 2H), as expected.

**TABLE 3:** Clinical and biological features of the TCGA cohort according to the presence of PTLS

Variables	Available data	TLS+ *n* (%)	*p* value
Age (years)			
>55	34	25(73.5%)	0.22
≤55	16	9(56.3%)	
Gender			
Male	28	22(78.6%)	0.07
Female	22	12(54.5%)	
HBV infection			
Yes	20	15(75%)	0.54
No	27	18(66.7%)	
HCV infection			
Yes	8	7(87.5%)	0.41
No	39	26(66.7%)	
AFP (ng/ml)			
>300	7	5(71.4%)	1
≤300	27	20(74.1%)	
TNM stage			
I/II	17	12(70.6%)	0.78
III/IV	33	22(66.7%)	

*Note*: Statistical analysis was performed using chi‐square tests.

Abbreviations: AFP, alpha‐fetoprotein; HBV, hepatitis B virus; HCV, hepatitis C virus; PTLS, peritumoral tertiary lymphoid structure; TCGA, the cancer genome atlas; TLS, tertiary lymphoid structure; TNM, tumor‐node‐metastasis.

**FIGURE 4:**
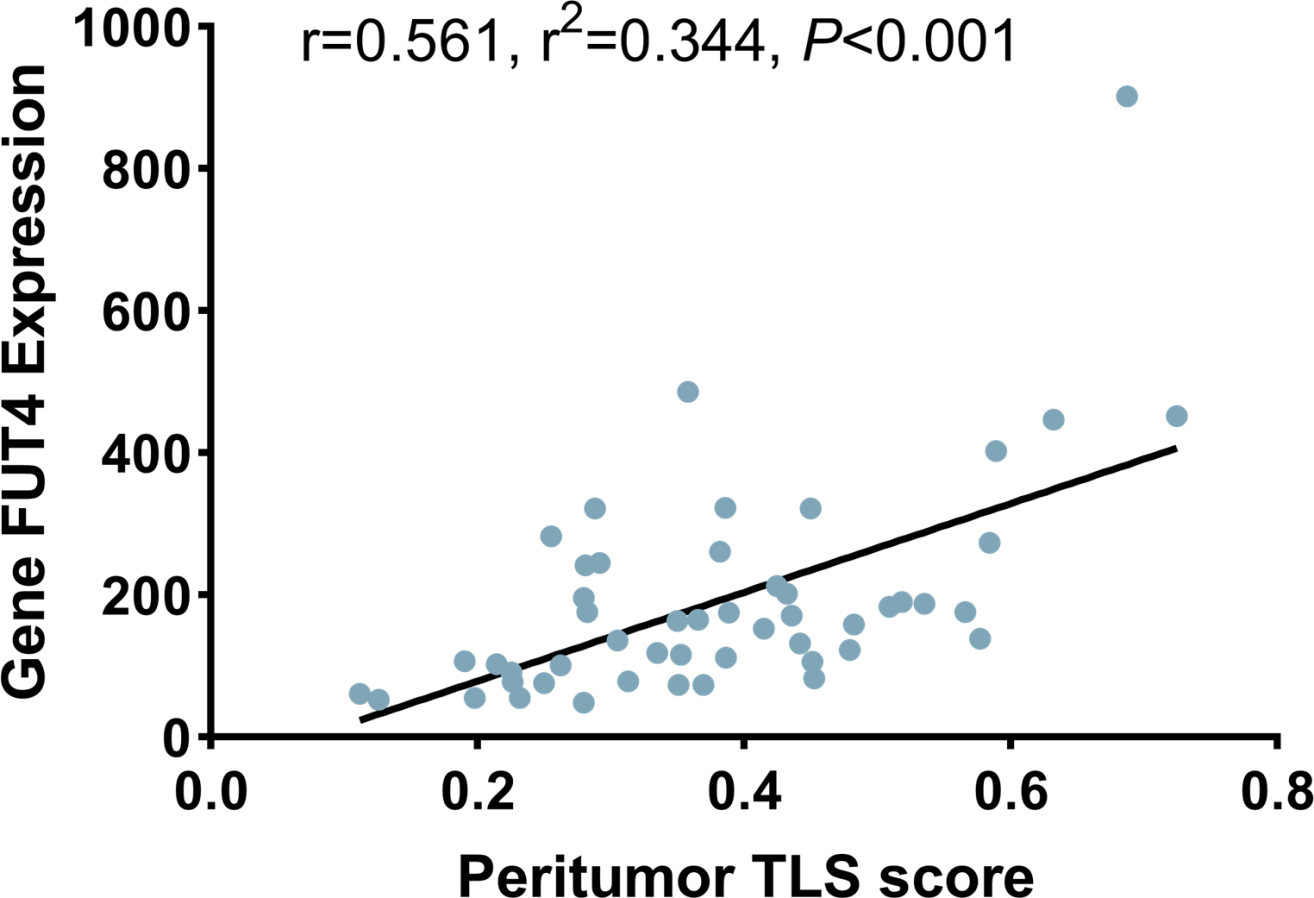
Linear relationship between peritumor TLS enrichment score and gene FUT4 expression.

To explore the circumstance of neutrophil infiltration in TCGA, gene FUT4 expression from the protein‐encoded transcriptomic data was used. Compared to the PTLS‐ group, higher expression of FUT4 in the PTS+ group in paracancer (PTLS− vs. PTLS+, 12.62, 30.03, *p* < 0.001), and the two groups in cancer (PTLS− vs. PTLS+, 23.28, 26.24, *p* = 0.54) were not statistically significant (Figure 3C). However, no statistical difference in FUT4 expression, with ITLS grouping, was found between peritumor (ITLS− vs. ITLS+, 24.78, 27.54, *p* = 0.56) and intratumor (ITLS− vs. ITLS+, 25.64, 25.12, *p* = 0.91) **(**Figure 3D). Correspondingly, the linear relationship between TLS and neutrophil infiltration was verified (Spearmen's *r* = 0.561, *r* ^2^ = 0.344, *p* < 0.001) (Figure 4), as both TLS score and FUT4 expression were continuous type variables.

## 4 DISCUSSION

TLS has been demonstrated in malignant tumors in terms of prognostic prediction value and immunotherapy unfolding in the clinic due to its specific spatial structure, which provides immune response sites for TME.^10^, ^11^, ^24^ Our work did find that PTLS represented a poor prognosis and showed a lower DFS as the total area of PTLS increased. We also confirmed that ITLS represented a good prognosis as in previous studies, but unfortunately did not find a stratification value of ITLS area class in prognosis. This may suggest dual prognostic and anti‐tumor or tumor‐promoting roles of TLS in distinct regions in HCC, a phenomenon also found in breast cancer^25^, ^26^ and intrahepatic cholangiocarcinoma (ICCA).^27^ In addition, we observed an increase in neutrophil infiltration with increasing grade of TLS in peritumor region of HCC. This may explain part of the association of PTLS with poor prognosis, as studies have shown that in malignant tumors, an increased number of neutrophils represents a worse survival.^28^, ^29^, ^30^ However, it is worth noting that the different relationship of TLS and neutrophil infiltration between intratumor and peritumor may indicate dual immune functions of TLS on the other hand.

In a present study, Ding GY et al. identified that Treg cells were significantly increased in intra‐tumor TLS than peri‐tumor counterpart, and its frequencies within ITLS were positively associated with abundance of PTLS in ICCA.^27^ The presence of Treg in TLS may negatively affect the levels of activation and infiltration of CD4+ and CD8 + T cells, resulting in tumor escape from immune surveillance.^31^ Sofopoulos M et al.^25^ discovered that patients with peritumoral TLS showed worse disease‐free survival (DFS) and overall survival (OS) as compared to patients lacking TLS. Moreover, the density of PTLS was also found to be crucial for prognosis, since patients with abundant TLS displayed the worst DFS and OS. Typically, Finkin et al.^16^ illustrated the earliest malignant hepatocyte progenitor cells appeared first within newly formed TLS and not elsewhere in the liver parenchyma. The findings further suggested that activation of the IκB kinase (IKK)–NF‐κB signaling pathway might be an important mediator of hepatic TLS generation. Knockout of adaptive immunity gene in IKKβ(EE)Hep mice found that TLS was completely eliminated,^16^ which could be used as a basis for targeted reduction of TLS.

In fact, TLS is a structure aggregated with abundance of immune cells like T and B cells mainly, also including other adaptive and innate immune cells. As a typical innate immune cell, neutrophils are the first line of defense of the body's immune defense.^32^, ^33^ Lymphocytes can recognize tumor cells and have the function of antagonizing tumor activity and preventing tumor cell proliferation and metastasis, while vascular endothelial growth factor (VEGF), matrix metalloproteases released from neutrophils can limit the immune function by inhibiting the killing effect of lymphocytes, resulting in the body in an immunosuppressed state, thus accelerating the proliferation and differentiation of tumor cells.^34^, ^35^, ^36^ In addition, granulocytic myeloid‐derived suppressor cells (G‐MDSC), a type of neutrophil, have been shown to have a strong immunosuppressive effect.^37^, ^38^ Macrophages in the TME are also typical innate immune cells, mainly involved in tumor progression and metastasis.^39^ M1 macrophages release nitric oxide (NO), reactive oxygen species (ROS), and proinflammatory cytokines interleukin (IL)‐1, IL‐6, IL‐12, TNF‐α, CXCL5, and CXCL8–10 to exert pro‐inflammatory functions, involving antigen processing and presentation, and promoting the function of effector T cells.^40^ M2 macrophages exert immunosuppressive functions and promote tissue repair by secreting IL‐10 and other immunosuppressive cytokines.^41^, ^42^ It is worth investigating whether different subtypes of macrophages are associated with different functions of TLS.

According to previous research, IL17 may explain the parallel increasing sights of TLS and neutrophils in peritumor. The production of various chemokines in TLS, notably CXCL12, CXCL13, CCL19, and CCL21, were induced by the combination of lymphotoxinβ (LTβ)–lymphotoxinβ receptor (LTβR) signaling pathway and IL‐17 secretion by lymphoid tissue inducer cells.^5^ Interestingly, Kuang DM et al. found that in liver cancer, IL17 induced neutrophil migration by upregulating the expression of chemokines such as CXCL1, CXCL2, CXCL3, CXCL5, CXCL8, and CXCL11.^29^ IL17 was produced by certain helper T cells (th17 cells) and is more widely distributed around cancer than within cancer.^43^

As the advantages of TLS in tumor immunotherapy have been demonstrated, studies on the induction and elimination of TLS have been carried out. There is evidence that an allogeneic vaccine can induce TLS aggregates after 2 weeks of treatment.^44^ Selective internal radiation therapy significantly promotes the recruitment of effector immune cells to take the shape of the TLS within the tumor.^45^ In addition, Immunostimulatory agonistic CD40 antibodies (αCD40) and CX3CR1+ macrophages have also been shown the ability to promote TLS formation.^46^, ^47^ In contrast, the TLS could be disrupted by LTβR blockade in vivo.^48^ Goodwin TJ et al. verified that lipid calcium phosphate nanoparticles containing the pCXCL12 trap resolved the formation of immune suppressive TLS.^49^

The advantage of the study is that for the first time, the total area metric was used to more comprehensively assess the distribution density of TLS, as TLS is a three‐dimensional structure, but common detection methods are based on the observation of two‐dimensional planar pathological sections. Similarly, DFS was used for the first time to explore the prognostic outcome of TLS in HCC, a frequent indicator for assessing the efficacy of disease treatment, as vaccine therapy for TLS is already being exploited. Perhaps, there are two forms of TLS present in HCC, a typical inflammation‐driven cancer with hepatitis and alcoholic steatohepatitis, as TLS formation is often in chronic inflammation and tumors. In a preneoplastic research, compared with surrounding cirrhotic nodules, all stages of early liver lesions showed increased densities of T cells, B cells, and dendritic cells in immature TLS.^50^ Therefore, it is necessary to further investigate which liver state induces the source of TLS formation. In this paper, the concept of peritumor is based on the tissue far away from the cancer edge, which is different from some previous studies in which the paracancer is only 1 mm away from the tumor margin, and it also has a certain focus on the junction area.^17^, ^27^ It is, noting that the different distance of peritumoral TLS from the cancer center may affect the formation and immune function of PTLS, which requires further research.

In addition, although the assessment of TLS by 12 chemokines has been widely used, there are fewer paired HCC data contained cancer and paracancer in the TCGA database as a validation cohort. This led to the validation of the prognostic impact of TLS in OS outcomes only, and PTLS was not an independent prognostic factor for OS by Cox multifactor regression analysis, as expected. In fact, PTLS is an inferior prognosis based on OS and DFS in training cohort by KM survival curve and COX analysis when using TLS−/TLS+ two groups, but it is a pity that only DFS makes sense when using TLS−/TLSL/TLSHs three groups. More evidence is needed to support the specific role of PTLS in different prognostic outcomes.

In conclusion, TLS remains an enigmatic immune compartment structure and opens up many new opportunities for cancer treatment. This paper clarifies the novel idea that the TLS in peritumor of HCC has a worse DFS with increasing density, and combined with previous studies, it illustrates the dual role of TLS in terms of prognosis and immunity in HCC. However, the mechanism remains unclear and further research is needed. Theoretically, the most favorable microenvironment may be achieved by promoting the formation of TLS within hepatocellular carcinoma and curbing its development in the peri‐cancerous area, which may be difficult to achieve realistically on target. Perhaps, it is essential to discover a medium that leads to the differentiation of TLS into different functions, neutrophils should be noted, which requires multicenter propensity‐matching analysis combining both intratumor and peritumor samples. In addition, the relationship between peritumor TLS and immune checkpoint inhibitors needs to pay attention.

## AUTHOR CONTRIBUTIONS

Tianchen Zhang, Xinjun Lei, Kaishan Tao, and Wenjie Song carried out study design. Tianchen Zhang, Xinjun Lei, Weili Jia, Jianhui Li, Ye Nie, Zhenzhen Mao, and Yanfang Wang were involved in acquisition of data. Tianchen Zhang, Xinjun Lei, and Wenjie Song carried out analysis of data. Tianchen Zhang, Xinjun Lei, and Weili Jia carried out statistical analysis. All authors were involved in critical revision of the manuscript.

## CONFLICT OF INTEREST

None.

## ETHICS APPROVAL STATEMENT

This study was approved by the Institutional Ethics Committee and informed consent was obtained prior to surgery for the use of surgical specimens and associated clinical data. (This phrase is in the Material & Methods section of the text. Ethical supporting information in the supplementary document named “ethics”).

## Supporting information


Table S1–S3

## Data Availability

Data available on request due to privacy/ethical restrictions: The data that support the findings of this study are available on request from the corresponding author. The data are not publicly available due to privacy or ethical restrictions.
