# Supplementary material for: Peritumor tertiary lymphoid structures are associated with infiltrating neutrophils and inferior prognosis in hepatocellular carcinoma
Source: Cancer Med. 2022 Sep 9;12(3):3068–78. doi: 10.1002/cam4.5227 (PMC9939159; doi:10.1002/cam4.5227)
Supplement: Supplementary file 1 — Table S1–S3 [file CAM4-12-3068-s001.docx]

| **Table S1. clinical and biological features of the XJ cohort according to the presence of ITLS.** | | | | | |  |
| --- | --- | --- | --- | --- | --- | --- |
| **Variables** | | **n=170** | **TLS- n=97(57%)** | **TLS_L_ n=36(21%)** | **TLS_H_ n=37(22%)** | **p value** |
| Age (years) | > 55 | 81 | 49(60.5%) | 15(18.5%) | 17(21%) | 0.64 |
|  | ≤ 55 | 89 | 48(53.9%) | 21(23.6%) | 20(22.5%) |  |
| Gender | Male | 143 | 81(56.6%) | 33(23.1%) | 29(20.3%) | 0.29 |
|  | Female | 27 | 16(59.3%) | 3(11.1%) | 8(29.6%) |  |
| Smoking | Yes | 64 | 39(60.9%) | 16(25%) | 9(14.1%) | 0.15 |
|  | No | 106 | 58(54.7%) | 20(18.9%) | 28(26.4%) |  |
| Alcohol | Yes | 33 | 18(54.5%) | 11(33.3%) | 4(12.1%) | 0.10 |
|  | No | 137 | 79(57.7%) | 25(18.2%) | 33(24.1%) |  |
| HBV infection | Yes | 127 | 79(62.2%) | 20(15.7%) | 28(22%) | 0.009 |
|  | No | 43 | 18(41.9%) | 16(37.2%) | 9(20.9%) |  |
| Cirrhosis | Yes | 113 | 66(58.4%) | 24(21.2%) | 23(20.4%) | 0.81 |
|  | No | 57 | 31(54.4%) | 12(21.1%) | 14(24.6%) |  |
| AFP (ng/ml) | > 300 | 59 | 30(50.8%) | 13(22%) | 16(27.1%) | 0.40 |
|  | ≤ 300 | 111 | 67(60.4%) | 23(20.7%) | 21(18.9%) |  |
| ALBI grade | I | 39 | 25(64.1%) | 9(23.1%) | 5(12.8%) | 0.30 |
|  | II/III | 131 | 72(55%) | 27(20.6%) | 32(24.4%) |  |
| TNM stage | I/II | 28 | 17(60.7%) | 6(21.4%) | 5(17.9%) | 0.85 |
|  | III/IV | 142 | 80(56.3%) | 30(21.1%) | 32(22.5%) |  |
| Tumor number | single | 25 | 16(64%) | 3(12%) | 6(24%) | 0.48 |
|  | mutiple | 145 | 81(55.9%) | 33(22.8%) | 31(21.4%) |  |
| Tumor size (cm) | > 5 | 91 | 61(67%) | 15(16.5%) | 15(16.5%) | 0.02 |
|  | ≤ 5 | 79 | 36(45.6%) | 21(26.6%) | 22(27.8%) |  |
| Differentiated degree | poor | 24 | 13(54.2%) | 2(8.3%) | 9(37.5%) | 0.07 |
|  | middle/high | 146 | 84(57.5%) | 34(23.3%) | 28(19.2%) |  |
| Capsular | Yes | 141 | 82(58.2%) | 32(22.7%) | 27(19.1%) | 0.16 |
|  | No | 29 | 15(51.7%) | 4(13.8%) | 10(34.5%) |  |
| MVI | Yes | 86 | 42(48.8%) | 20(23.3%) | 24(27.9%) | 0.07 |
|  | No | 84 | 55(65.5%) | 16(19%) | 13(15.5%) |  |
| Statistical analysis was performed using chi-square tests. PTLS, peritumoral tertiary lymphoid structure; HBV, hepatitis B virus; AFP, alpha-fetoprotein; ALBI, albumin-bilirubin; TNM, tumor-node-metastasis; MVI, microvascular invasion. | | | | | | |
|  |  |  |  |  |  |  |

| **Table S2. clinical and biological features of the NEW XJ cohort PTLS for neutrophil** | | | | | |  |
| --- | --- | --- | --- | --- | --- | --- |
| **Variables** | | **n=85** | **TLS- n=14(17%)** | **TLS_L_ n=37(44%)** | **TLS_H_ n=34(39%)** | **p value** |
| Age (years) | > 55 | 47 | 11(23.4%) | 19(40.4%) | 17(36.2%) | 0.07 |
|  | ≤ 55 | 38 | 3(7.9%) | 18(47.4%) | 17(44.7%) |  |
| Gender | Male | 69 | 10(14.5%) | 28(40.6%) | 31(44.9%) | 0.15 |
|  | Female | 16 | 4(25%) | 9(56.3%) | 3(18.8%) |  |
| Smoking | Yes | 29 | 5(17.2%) | 9(31%) | 15(51.7%) | 0.18 |
|  | No | 56 | 9(16.1%) | 28(50%) | 19(33.9%) |  |
| Alcohol | Yes | 15 | 4(26.7%) | 3(20%) | 8(53.3%) | 0.09 |
|  | No | 70 | 10(14.3%) | 34(48.6%) | 26(37.1%) |  |
| HBV infection | Yes | 61 | 10(16.4%) | 25(41%) | 26(42.6%) | 0.55 |
|  | No | 24 | 4(16.7%) | 12(50%) | 8(33.3%) |  |
| Cirrhosis | Yes | 55 | 8(14.5%) | 22(40%) | 25(45.5%) | 0.37 |
|  | No | 30 | 6(20%) | 15(50%) | 9(30%) |  |
| AFP (ng/ml) | > 300 | 32 | 4(12.5%) | 13(40.6%) | 15(46.9%) | 0.59 |
|  | ≤ 300 | 53 | 10(18.9%) | 24(45.3%) | 19(35.8%) |  |
| ALBI grade | I | 20 | 3(15%) | 7(35%) | 10(50%) | 0.55 |
|  | II/III | 65 | 11(16.9%) | 30(46.2%) | 24(36.9%) |  |
| TNM stage | I/II | 12 | 2(16.7%) | 5(41.7%) | 5(41.7%) | 0.97 |
|  | III/IV | 73 | 12(16.4%) | 32(43.8%) | 29(39.7%) |  |
| Tumor number | single | 13 | 2(15.4%) | 4(30.8%) | 7(53.8%) | 0.50 |
|  | mutiple | 72 | 12(16.7%) | 33(45.8%) | 27(37.5%) |  |
| Tumor size (cm) | > 5 | 42 | 5(11.9%) | 20(47.6%) | 17(40.5%) | 0.68 |
|  | ≤ 5 | 43 | 9(20.9%) | 17(39.5%) | 17(39.5%) |  |
| Differentiated degree | poor | 12 | 3(25%) | 7(58.3%) | 2(16.7%) | 0.19 |
|  | middle/high | 73 | 11(15.1%) | 30(41.1%) | 32(43.8%) |  |
| Capsular | Yes | 69 | 8(11.6%) | 30(43.5%) | 31(44.9%) | 0.06 |
|  | No | 16 | 6(37.5%) | 7(43.8%) | 3(18.8%) |  |
| MVI | Yes | 51 | 7(13.7%) | 24(47.1%) | 20(39.2%) | 0.83 |
|  | No | 34 | 7(20.6%) | 13(38.2%) | 14(41.2%) |  |
| Statistical analysis was performed using chi-square tests. PTLS, peritumoral tertiary lymphoid structure; HBV, hepatitis B virus; AFP, alpha-fetoprotein; ALBI, albumin-bilirubin; TNM, tumor-node-metastasis; MVI, microvascular invasion. | | | | | | |
|  |  |  |  |  |  |  |

| **Table S3. Analysis of OS in TCGA cohort** | | | | | |
| --- | --- | --- | --- | --- | --- |
|  | **Univariate analysis** | |  | **Multivariate analysis** | |
| **Variables** | **HR(CI 95%)** | ***p* value** |  | **HR(CI 95%)** | ***p* value** |
| Age > 55 years | 0.72(0.35-1.51) | 0.38 |  |  |  |
| Male sex | 4.02(1.69-9.55) | 0.002 |  | 2.96(1.2-7.31) | 0.02 |
| HBV infection | 0.93(0.47-1.87) | 0.84 |  |  |  |
| HCV infection | 1.56(0.67-3.61) | 0.30 |  |  |  |
| AFP > 300 (ng/ml) | 0.62(0.21-1.85) | 0.39 |  |  |  |
| Tumor stage (III/IV) | 1.35(0.67-2.72) | 0.40 |  |  |  |
| TLS+ | 3.35(1.35-8.34) | 0.009 |  | 2.36(0.88-6.29) | 0.08 |
| Statistical analysis was performed using univariate and multivariate Cox proportional hazards regression models. OS, overall survival; HBV, hepatitis B virus; AFP, alpha-fetoprotein; TLS, tertiary lymphoid structure; HR, hazard ratio. | | | | | |
